# Imaging of COVID-19 vasculopathy from head to toe: Egyptian collective experience after 2 years of the pandemic

**DOI:** 10.1186/s43055-022-00815-y

**Published:** 2022-06-21

**Authors:** Ahmed Fathy, Adel Rizk, Abdelaziz Elnekeidy, Heba Said Gharraf, Mohamed Saied Abdelgawad, Ahmed Samir

**Affiliations:** 1 Department of Radio-Diagnosis, Faculty of Medicine, Alexandria University, Alexandria, Egypt; 2 Department of Chest Diseases, Faculty of Medicine, Alexandria University, Alexandria, Egypt; 3 Department of Radio-Diagnosis and Intervention, National Liver Institute, University of Menoufia, Shibin El Kom, Egypt

**Keywords:** Covid-19, Vascular, Vasculopathy, Imaging, Egyptian

## Abstract

**Background:**

COVID-19 vasculopathy is a critical condition that impacts the disease prognosis including vasculitis and thromboembolic complications. This study aimed to provide the Egyptian experience about the COVID-19 vasculopathy during the past two years of the pandemic and to collectively include the different modalities and imaging techniques for the diagnosis of cerebrovascular, pulmonary, gastrointestinal, and peripheral arterial vascular complications.

**Results:**

This is a multi-center retrospective analysis of 3500 PCR-proved COVID-19 infection between March 2020 and December 2021. A cohort of 282 consecutive patients with COVID-19 vasculopathy was considered for inclusion. They included 204 males and 78 females (72:28%). The mean age was 68 years, and age ranged from 48 to 90 years. Five radiologists evaluated the different imaging examinations in consensus including computed tomography (CT), CT-angiography (CTA), CT-perfusion (CTP), magnetic resonance imaging (MRI), MR-arteriography (MRA), and MR-venography (MRV). 244/282 (86.5%) patients suffered from non-hemorrhagic cerebral ischemic infarctions. 13/282 (4.6%) patients suffered from hemorrhagic cerebral infarctions. 5/282 (1.8%) patients suffered from cerebral vasculitis. Pulmonary vascular angiopathy was detected in 10/282 (3.5%) patients, including pulmonary embolism in 10/10 patients, pulmonary infarctions in 8/10 patients, pulmonary vascular enlargement in 5/10 patients, and vascular "tree-in-bud" sign in 2/10 patients. Intestinal ischemia and small bowel obstruction were detected in 3/282 patients (1%) while GIT bleeding was encountered in 4/282 patients (1.4%). Lower limb arterial ischemia was found in 3/282 patients (1%). Additionally; 39/282 (13.8%) patients developed peripheral deep venous thrombosis (DVT) due to prolonged ICU recumbence while 28/282 (10%) patients developed jugular vein thrombosis sequel to prolonged catheterization. A *p* value (0.002) and (*r*) = 0.8 statistically proved strong significant relation between COVID-19 vasculopathy and D-dimer levels.

**Conclusions:**

Multi-system vasculopathy was a serious complication of COVID-19 which impacted the patients' morbidity and mortality. An Egyptian experience about the COVID-19 vasculopathy during the past two years of the pandemic was provided. It encountered the different modalities and imaging techniques for the diagnosis of cerebrovascular, pulmonary, gastrointestinal, and peripheral arterial COVID-19 vascular complications.

## Background

COVID-19 vasculopathy is a critical condition that impacts the disease prognosis including vasculitis and thromboembolic complications [1]. It is considered the most common non-respiratory complication of COVID-19 infection which is encountered in 31% of COVID-19 patients in the intensive care units (ICU) [2, 3].

Two different pathophysiologic mechanisms can explain the development of multi-systemic COVID-19 vasculopathies. The first mechanism is the high levels of blood cytokines including the interleukin-6 (IL-6), which is referred to as "the cytokine storm" with thrombin generation sequel to the tissue factor expression on the macrophages and the monocytes. The second mechanism is the vascular inflammatory response or vasculitis which occurs due to direct infection of the endothelial cells and expression of ACE2 [4–6].

Cerebro-vascular infarcts are frequent in COVID-19 patients with or without venous thrombosis, disruption of the blood–brain barrier, and secondary hemorrhagic transformation [7, 8].

In absence of severe lung involvement, the development of adult respiratory distress syndrome (ARDS) could be attributed to pulmonary vasculopathy, endothelial damage, and pulmonary embolism (PE) [9].

Acute intestinal ischemia and GIT bleeding are also considered rare vascular complications of COVID-19 infection which can complicate porto-mesenteric venous thrombosis or inflammatory bowel disorders [10].

Additionally, peripheral limb ischemia is a rare COVID-19 vascular complication with dysfunction of the coagulation mechanisms [11].

This study aimed to provide the Egyptian experience about the COVID-19 vasculopathy during the past two years of the pandemic and to collectively include the different modalities and imaging techniques for the diagnosis of cerebrovascular, pulmonary, gastrointestinal, and peripheral arterial vascular complications.

## Methods

### Study population and ethical protocol

This is a multi-center retrospective analysis of 3500 PCR-proved COVID-19 infection between March 2020 and December 2021. A cohort of 282 consecutive patients with COVID-19 vasculopathy was considered for inclusion.

Inclusion criteria were as follows: (1) Patients with a positive PCR test for Covid-19 infection, (2) Patients with available full clinical and laboratory records, and (3) Patients with routine chest CT and a positive imaging examination for COVID-19 vasculopathy.

Exclusion criteria were as follows: (1) Past history of vascular occlusion which necessitated seeking medical advice or primary vascular diseases such as vasculitis, (2) History of demyelinating diseases which may mimic the picture of neuro-vasculitis, (3) Incomplete medical records, and (4) Poor quality of images.

The Ethics Committee at the authors’ institution approved the study. On a retrospective basis, the Research Ethics Board waived the need for informed patient consent, stressing the assurance of the confidentiality of the patients' information and the medical records.

The patient cohort comprised 282 patients (204 males and 78 females, mean age 68 years ± 9 SD, age range 48–90 years).

A single expert consulting pulmonologist shared in this study with 21 years of experience in the field of pulmonology intensive care units.

The different imaging studies were analyzed in consensus by four expert consulting radiologists (14–33 years of experience) in addition to a senior resident.

### Imaging machines

- Two types of MDCT scanners were utilized; the first is SOMATOM Sensation 64 (Siemens, Erlangen, Germany), while the other is Aquilion CXL/CX 128 (Toshiba, Canon Medical Systems, Tustin, CA, USA).
- Two types of 1.5-Tesla MRI scanners were utilized; the first is GE Medical Systems, Sigma, USA, while the other is Magnotom ESPREE 1.5 T, Erlangen, Semines, Germany.
- TOSHIBA Aplio 500 machine was utilized for Duplex assessment, using a transducer with frequency ranging from 5 to 13 MHz.

### Routine chest CT analysis

The typical pulmonary CT findings for COVID-19 infection were assessed including ground-glass opacities (GGOs) and consolidations. Quantitative volumetric assessment for the pathological lung parenchyma was estimated by OsiriX MD 11.0 software after ROI 2D/3D reconstruction and threshold interval adjustment. An international volumetric CT severity scoring was utilized as follows: Score 1 corresponds to 0–25% lung involvement, Score 2 corresponds to 26–50%, Score 3 corresponds to 51–75%, and Score 4 corresponds to > 75% lung involvement [12].

### Neurologic imaging analysis

Different imaging tools were included, as follows:

1. Non-enhanced CT for the brain was evaluated for diagnosis and localization of either non-hemorrhagic or hemorrhage ischemic infarctions as well as vasculitic areas.
2. CT-arteriography (CTA) of the cerebral circulations was utilized for diagnosis and localization of cerebrovascular arterial occlusion. It plays an important role for mapping prior to interventional procedures.
3. CT-perfusion (CTP) was utilized for delineation of the ischemic area as well as the cerebral blood flow and volume (CBF and CBV). A 60-s cine series was achieved at 0 to 5 s following injection of 40 ml of intra-venous non-ionic contrast at a 4–5 ml/s injection rate. CBV mapping was performed for the identification of hypo-perfused areas and the areas of the penumbra to predict patient prognosis and also as a base line examination before interventional procedure and follow up.
4. The conventional MRI sequences were utilized as follows: Axial and sagittal T1-WI, axial and coronal T2-WI, as well as FLAIR-WI. MRI examinations were requested by clinicians for either patients with unremarkable CT results such as in patients with vasculitis or patients with non-conclusive CT results as senile deep white matter lesions without definite CT signs of recent ischemia, or for confirmations of positive data in patients with small or subtle infarctions.
5. Diffusion-weighted image (DWI) MRI axial plane analysis was utilized. It is a T2-weighted, echo-planar spin-echo sequence EPI having MR-parameters as follows: TR = 3400 ms, TE = 100 ms, acquisition matrix = 192 × 192, slice thickness = 5 mm, gap = 0.3 mm with *b* value = 0, 500, 1000 s/mm^2^. The ADC value was measured in the ADC mapping inside the pathological area using a region of interest (ROI). Bright signal in the DWI in addition to low ADC value denotes positive diffusion restriction for recent ischemic infarctions.
6. Susceptibility weighted image (SWI) was utilized in addition to the conventional MRI sequences for detection of hemorrhagic transformation.
7. MR venography (MRV) was utilized via a time-of-flight (TOF) technique for the localization of venous sinus thrombosis. MRV examination were requested mainly in patients with infarctions along the water-shed zones especially when hemorrhagic.
8. MR arteriography (MRA) was utilized for mapping for large vascular occlusion prior to interventional procedures.

### Pulmonary CTA analysis

The examination was performed in a caudo-cranial direction using a single breath-hold with full inspiration in a supine position. The following parameters were used: kVp = 120, mAs = 50–100, slice thickness = 1.25 mm, FOV = 350 × 350. A 50–60 ml of non-ionic IV was injected at a flow rate of 5.0–6.0 ml/s followed by a saline chaser. A bolus tracking was used at the threshold of 100 HU on the pulmonary trunk.

Three different greyscale windows were utilized as follows: (1) the lung window at 1500/−600, (2) the mediastinal window at 350/40, and (3) the pulmonary embolism window at 700/100. Minimal intensity projection (MIP) volume reconstruction was utilized for the assessment of vascular structures.

The CT signs of pulmonary vasculopathy were assessed as follows:

- Acute pulmonary embolism is manifested by a central contrast filling defect.
- Wedge-shaped sub-pleural pulmonary infarct.
- Pulmonary vascular enlargement is manifested by abnormal pulmonary arterial dilatation inside and/or outside the pathological lung parenchyma with the loss of normal tapering [13].
- The vascular ''tree-in-bud'' sign is manifested by the beaded appearance of peripheral pulmonary arterioles [14].

### Gastro-intestinal CTA analysis

Urgent CTA examination, without special bowel preparation, was performed for assessment of the abdominal aorta and its major branches as well as the porto-mesenteric venous axis.

Patients with a clinical diagnosis of intestinal obstruction did not receive water oral intake or enema. On the other hand, patients with GIT bleeding received water enema without oral water intake because of severe vomiting.

Pre and post-contrast CT scans were performed. Post-contrast scans were obtained at the late arterial phase (45 s) and portal venous phases (70 s). Non-ionic 350 mol IV contrast medium was injected (1.5 ml/kg) at a flow rate of 3–4 ml/s.

Images were assessed using multi-planar reconstruction (MPR), curved planar reconstruction (CPR), and MIP.

- The examined arteries and veins were traced for detection of any filling defect of thrombosis.
- The small and large bowel were examined regarding the following: (1) Caliber: to diagnose and locate the site of obstruction, (2) Mural enhancement: to detect ischemic changes, (3) Submucosal edema (water halo sign), (4) Mural air (pneumatosis intestinal), (5) Surrounding fat stranding and (6) Loco-regional or distant free or loculated fluid collection.

### Peripheral arterial analysis

Duplex studies were utilized for the detection of peripheral or central deep venous thrombosis. While CTA of the lower limbs was performed for detection of acute arterial occlusion. The patient was in a supine position with his feet entering the gantry first. The non-contrast phase was initially obtained followed by the post-contrast early arterial and late venous runoff phases. IV contrast dose was 1.5 ml/kg. The acquisition parameters were as follows: gantry rotation = 400 ms, collimation = 64 × 10 mm, mA = 80, kV = 120.

### Statistical analysis

Mean, mode, median, variance, standard deviations, and ratios were calculated and compared. Chi-square and *p* value analysis was performed using an online calculator (http://www.alcula.com/calculators/statistics/). A *p* value < 0.05 was taken as a landmark for statistical significance. Additionally, a Pearson correlation coefficient (*r*) was utilized.

## Results

The majority of patients were in the sixth decade of life. The male to female ratio was 72%:28%. Hypertension was the most common comorbidity in 45% of patients. Cardiomegaly incidentally noted in 30% of patients. The D-dimer levels were high in 273/282 (97%). A *p* value (0.002) and (*r*) = 0.8 statistically proved strong significant relation between COVID-19 vasculopathy and D-dimer levels. 97% of the included patients presented during the first month of COVID-19 infection with shooting D-dimer levels and persistently positive PCR results. This clinical scenario, in addition to the absence of past history of serious vascular disease or other clinical explanation, put COVID-19 vasculopathy as the most reasonable scenario. The distribution of patients according to their clinical characteristics is detailed in (Table 1)*.*

**Table 1:** Summary of clinical data in each group of patients with COVID related vasculopathy

+Clinical parameters			Neuro-vasculopathy (262/282)		Pulmonary vasculopathy (10/282)		GIT vasculopathy (7/282)		Peripheral limb vasculopathy (3/282)	
[I]	Age									
	–	41–50 years	7 (3%)		–		–		–	
	–	51–60 years	43 (16%)		–		4 (57%)		2 (67%)	
	–	61–70 years	192 (73%)		9 (90%)		3 (43%)		1 (33%)	
	–	71–80 years	14 (5%)		1 (10%)		–		–	
	–	81–90 years	6 (2%)		–		–		–	
[II]	Sex									
	–	Male	188 (72%)		10 (100%)		3 (43%)		3 (100%)	
	–	Female	74 (28%)		–		4 (57%)		–	
[III]	Chest symptoms									
	–	Present	247 (94%)		10 (100%)		6 (86%)		2 (67%)	
	–	Absent (initial presentation by vasculopathy)	15 (6%)		–		1 (14%)		1 (33%)	
[IV]	Time of clinical presentation from onset of chest disease									
	–	1st week	102 (39%)		–		–		–	
	–	2nd week	111 (42%)		5 (50%)		3 (43%)		1 (33%)	
	–	3rd week	23 (9%)		3 (30%)		3 (43%)		1 (33%)	
	–	4th week	6 (2%)		2 (20%)		–		–	
	–	2nd month	3 (1%) (vasculitis)		–		–		–	
	–	3rd month	2 (1%) (vasculitis)		–		–		–	
[V]	Main complaint		DLC	257 (98%)	Fever	7 (70%)	Abd. Pain	7 (100%)	Leg pain:	3 (100%)
			Weakness/paresis	243 (93%)	Cough	10 (100%)	Vomiting	7 (100%)		
			Headache	5 (2%)	Dyspnea	10 (100%)	Hematemesis	4 (57%)		
			Dysarthria	4 (2%)	Chest pain	10 (100%)	Melena	4 (57%)		
			Vertigo	3 (1%)			Constipation	3 (43%)		
			Tinnitus	2 (1%)			Diarrhea	4 (57%)		
			Blurred vision	2 (1%)						
[VI]	Comorbidities									
	–	Diabetes	24 (9%)		3 (30%)		2 (28%)		1 (33%)	
	–	Hypertension	120 (46%)		3 (30%)		3 (43%)		2 (67%)	
	–	Cardiomegaly (incidental)	78 (30%)		2 (20%)		4 (57%)		2 (67%)	
	–	Hepatic disease	18 (7%)		–		–		–	
	–	Renal disease	28 (11%)		–		–		–	
	–	Neoplastic	9 (3%)		–		–		–	

**Fig. 1:**
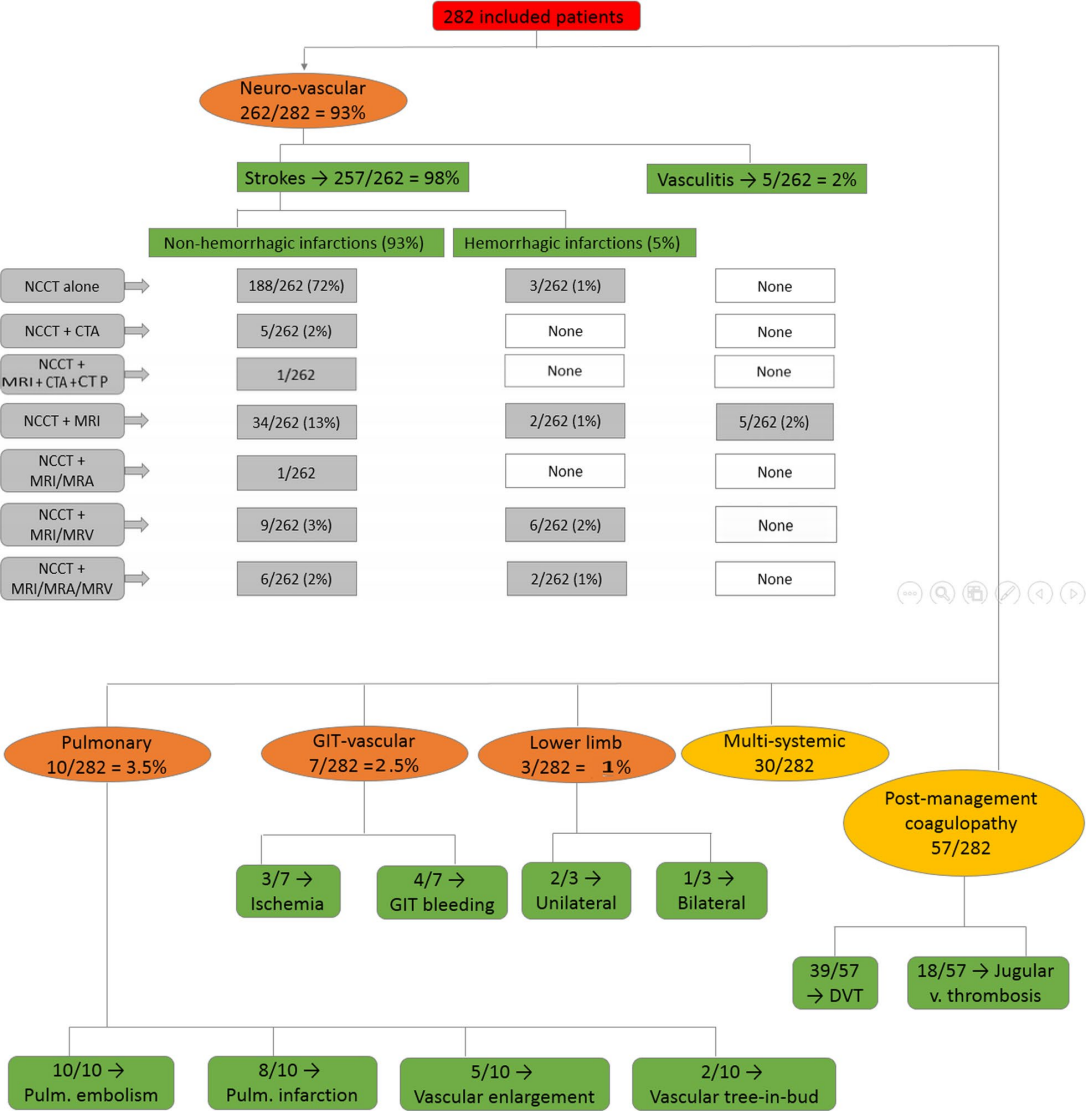
A flowchart is summarizing the incidence of COVID-19 vasculopathies

A chart summarizing the incidence of the COVID-19 vascular complications is demonstrated in Fig. 1.

### Neurologic and cerebrovascular results

Cerebro-vascular complications dominated in 262/282 (93%) of included patients*.* They were categorized as follows: 244/262 (93%) patients suffered from non-hemorrhagic cerebral ischemic infarctions, 13/262 (5%) patients suffered from hemorrhagic cerebral infarctions, and 5/262 (2%) patients suffered from cerebral vasculitis.

#### Clinically (Table 1)

The cerebrovascular thromboembolic complications mainly presented during the first and second weeks of infection (39% and 42% respectively), meanwhile 6% of patients first neurologically presented without chest symptoms.

On the other hand, cerebral vasculitis presented either two or three months after primary infection. Their probable diagnosis with correlation to COVID-19 was however clinically established based on the mild partially self-limited behavior of the disease during absence of past history of vasculitis or other reasonable explanation.

Around 92% of patients clinically presented with a disturbed level of consciousness and 93% of patients clinically presented with limb weakness up to hemiparesis. Vertigo, tinnitus, and blurring of vision were reported in patients with vasculitis.

#### Radiologically

**Fig. 2:**
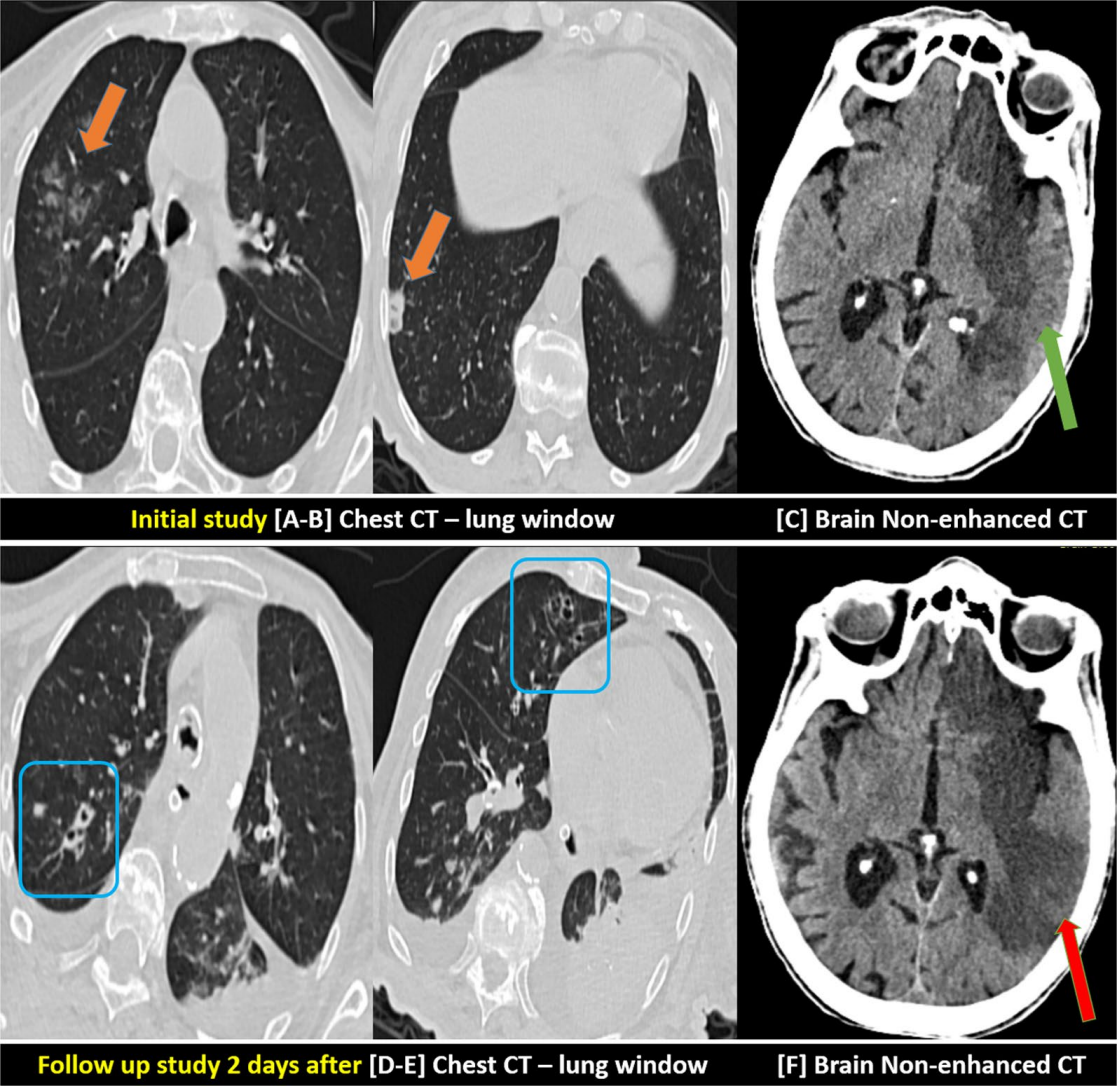
An 85-years-old female patient with COVID-19 infection complicated by invasive aspergillosis and cerebrovascular stroke: **A–C** Initial study: **A**, **B** Axial chest CT images (lung window) showing confluent right upper lobar ground-glass patches and right basal sub-pleural small cavitating consolidation (orange arrows). **C** Axial brain CT image showing large left fronto-tempro-pareital recent hypo-dense infarction along with the territory of the left ACA and MCA (partially sparing left temporal cortical area … green arrow). **D–F** Follow-up study after 2 days: **D**, **E** Axial chest CT images (lung window) showing denovo bilateral pleural collection and cavitating lung nodules (blue squares). **F** Axial brain CT image showing size progression of the previous large infarction involving more volume of the left temporal lobe (red arrow)

**Fig. 3:**
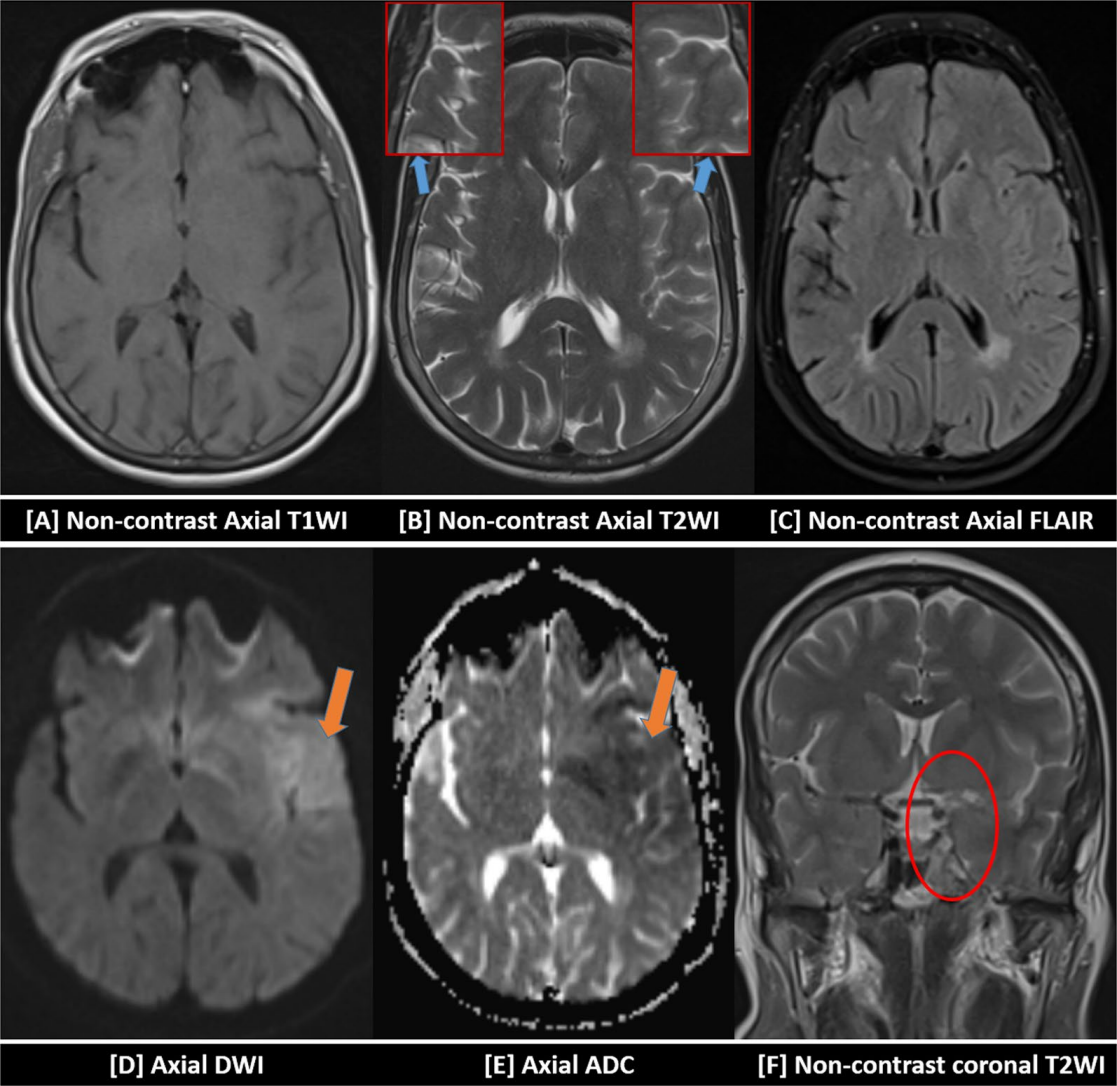
A-63 years-old female patient, with a history of cardiac disease, complained of disturbed level of consciousness and right hemiparesis two hours before visiting the emergency unit. No chest symptoms were reported. Urgent non-contrast brain CT was unremarkable on admission. Brain MRI was performed after two hours. Hypo-intense signal was noticed at the left temporal cortical and subcortical white matter in T1-WI [**A**]. A subtle cortical hyper-intense signal was barely noticed in T2-WI [**B**] and FLAIR-WI [**C**]. Positive diffusion restriction was noted in the form of a bright DWI signal [**D**] and a corresponding low ADC value in the ADC mapping [**E**] (orange arrows). Coronal T2-WI revealed non-visualization of the signal voids of the left ICA and MCA (red circle) [**F**] The initial diagnosis was a recent ischemic infarction along the left MCA territory

**Fig. 4:**
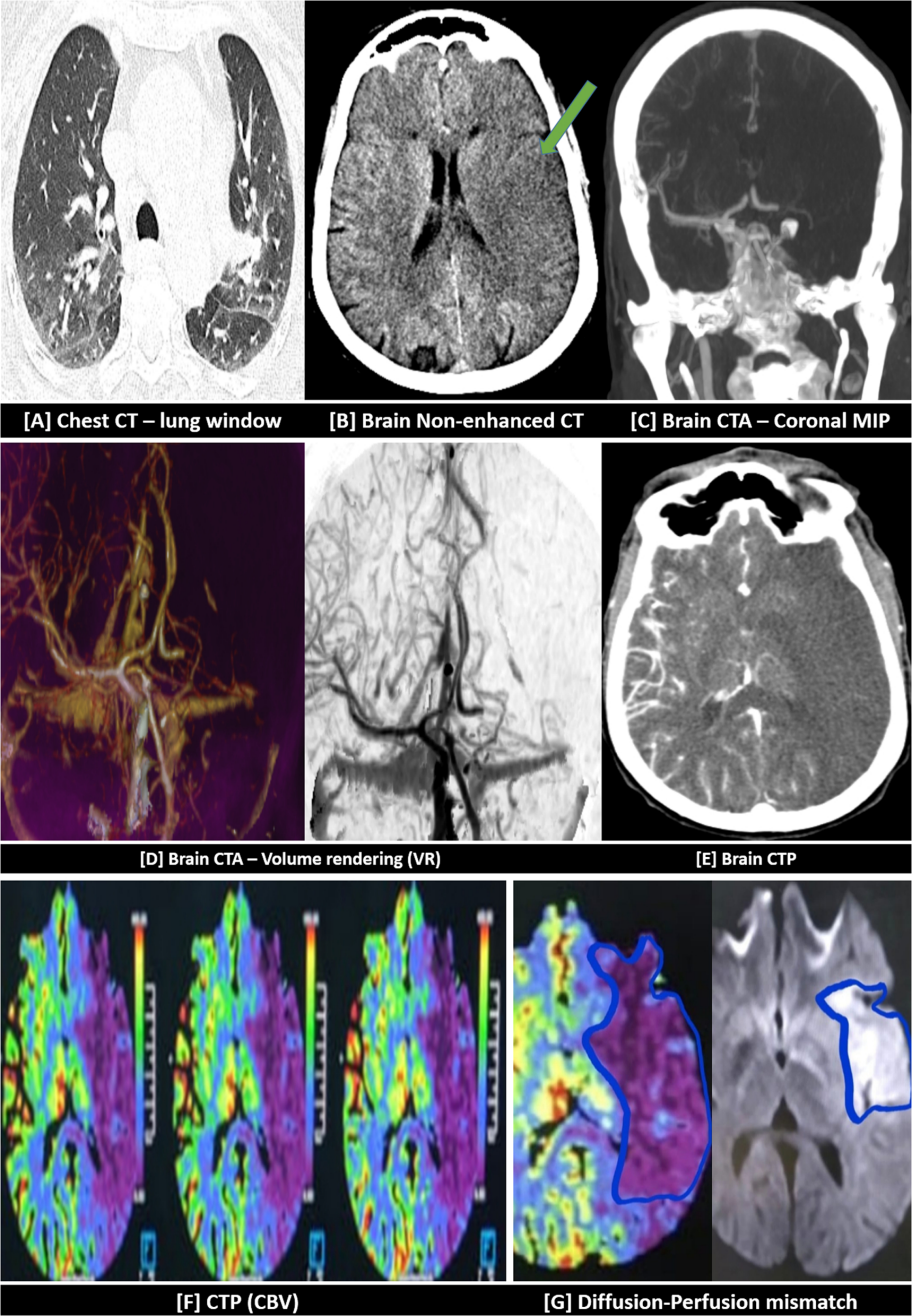
The above-mentioned patient in (Fig. 3) performed chest CT as well as brain CTA and CTP two hours later. [**A**] Chest CT image (lung window) revealed bilateral basal ill-defined ground-glass patches (suspicious for COVID-19 infection). [**B**] Initial non-contrast brain CT images revealed a large left frontotemporal, tempro-parietal, and tempro-occipital hypo-dense area (green arrow) implicating the cortical and subcortical white matter and extending to the left basal ganglia with effacement of the cortical sulci and left Sylvian fissure. CTA coronal MIP image [**C**] and volume rendering image [**D**] showed total occlusion and non-opacification of the intracranial segment of the left ICA and left MCA. Non-perfusion of this large area was noted in dynamic CT-perfusion [**E**, **F**]. An obvious diffusion-perfusion mismatch was noted denoting significant penumbra [**G**]. PCR test result was revealed to be positive for COVID-19

**Fig. 5:**
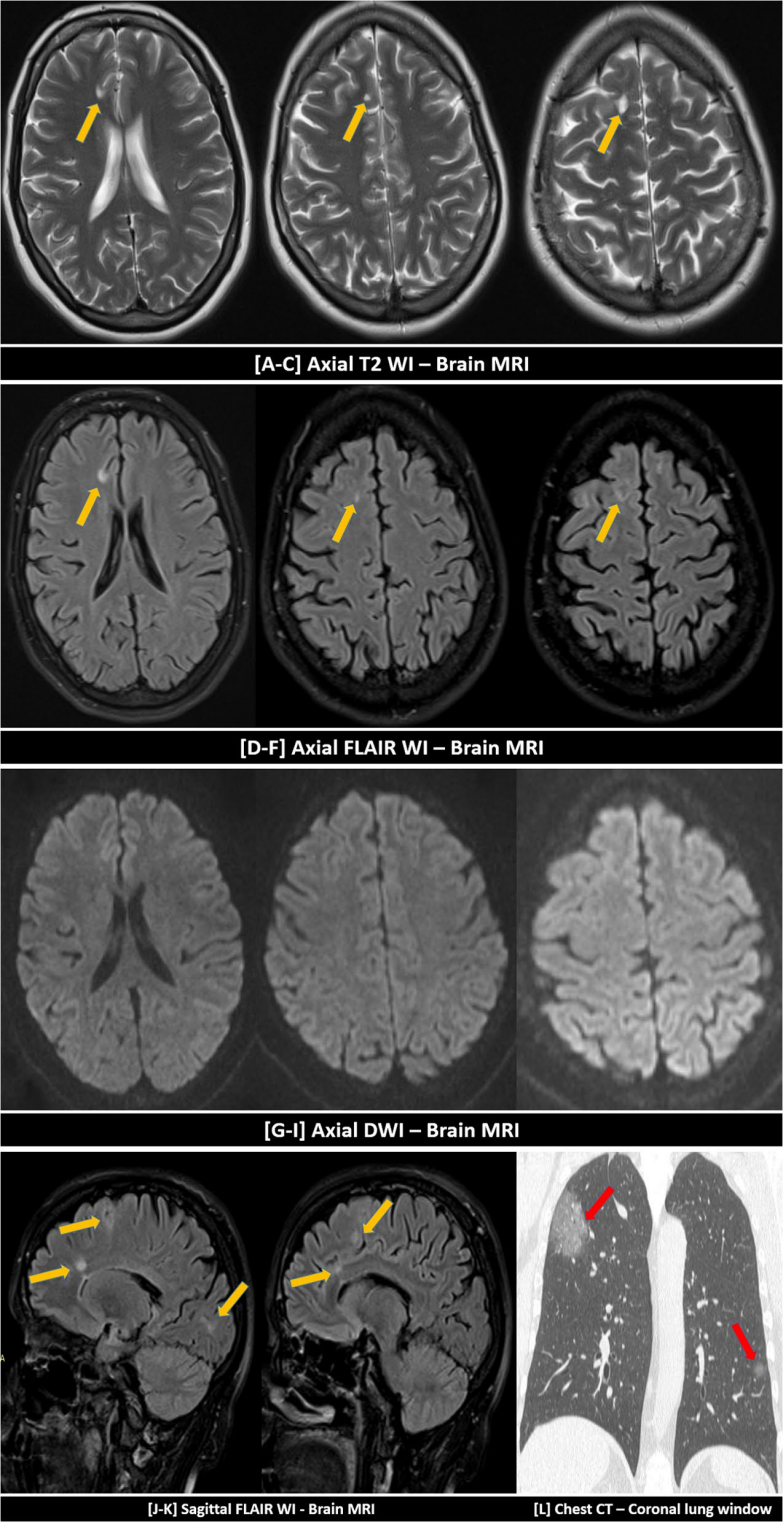
A 39-years-old female patient, with a history of COVID-19 infection two months ago, complained of dizziness. Partial improvement was achieved on supportive treatment. MRI was requested because of persistent symptoms. No previous history of neurological diseases. The neurological examination was free. Multiple small cortical and to a lesser extent sub-cortical as well as deep white matter lesions are seen in the right frontoparietal lobes (yellow arrows) showing: T2 hyper-intense signal [**A**–**C**], FLAIR hyper-intense signal [**D**–**F**] without DWI restriction [**G**–**I**]. [**J**, **K**] Sagittal FLAIR weighted images are demonstrating the lesions. [**L**] Coronal chest CT (lung window) 2 months ago showed a right apical sub-pleural ground-glass patch of COVID-19 infection. Symptoms alleviated after a short course of steroid therapy with resolution of MRI lesions… both, the clinical context and cortical involvement, suggested the possibility of post-COVID vasculitis rather than demyelinating diseases

**Fig. 6:**
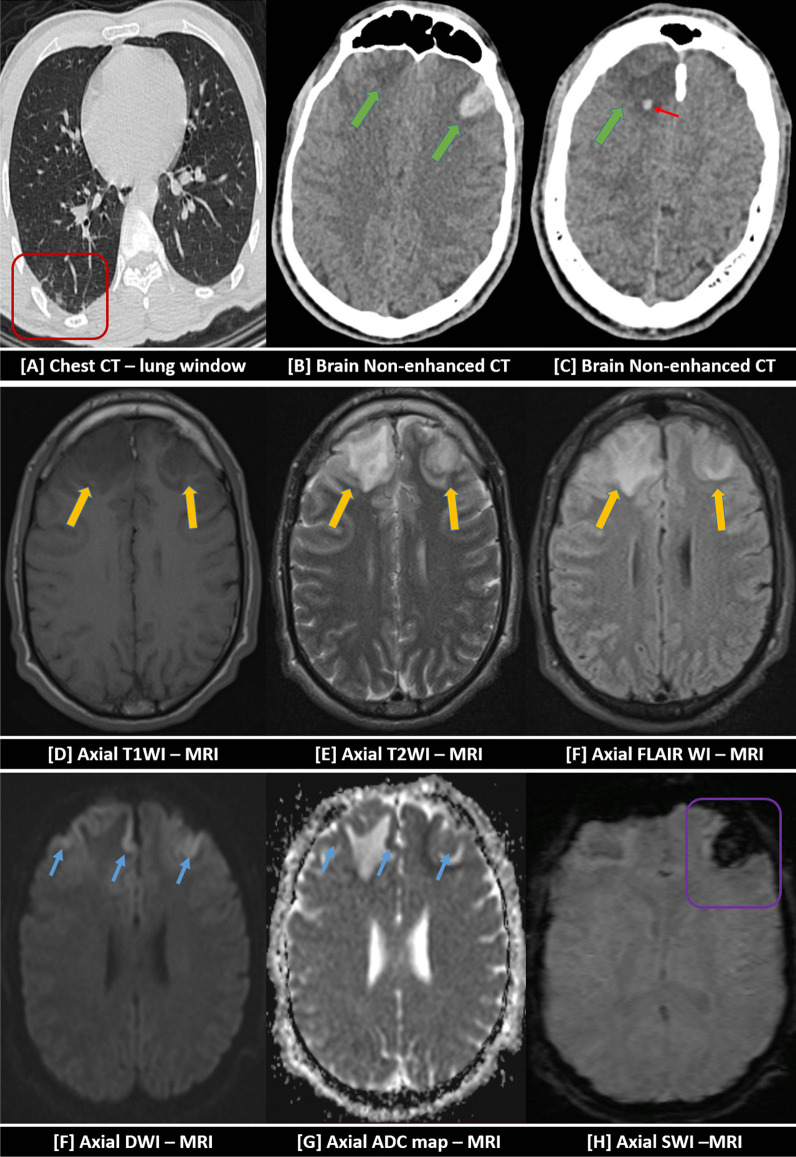
An 84-years-old female patient was proved for COVID-19 infection after one week of cough and mild fever. She presented to the emergency unit with a suddenly disturbed level of consciousness. [**A**] Chest CT examination (lung window) revealed small right basal sub-pleural ground-glass patches of COVID-19 infection (red square). [**B**, **C**] Urgent non-contrast brain CT revealed bilateral high frontoparietal cortical and sub-cortical heterogeneous mixed hypo and hyper-dense areas (green and red arrows) … The initial diagnosis was bilateral hemorrhagic infarctions. An initial non-contrast MRI examination was performed. They express heterogeneous hypointense signals in T1-WI [**D**] and mixed iso and hyperintense signals in T2-WI [**E**] and FLAIR-WI [**F**] (yellow arrows). Positive diffusion restriction was noted with cortical bright DWI signal [**G**] and low ADC value in ADC mapping [**G**] (blue arrows). Hemorrhagic transformation was evidenced by positive SWI signal blooming [**H**] (violet square) … Hyper-acute hemorrhagic transformation was diagnosed

**Fig. 7:**
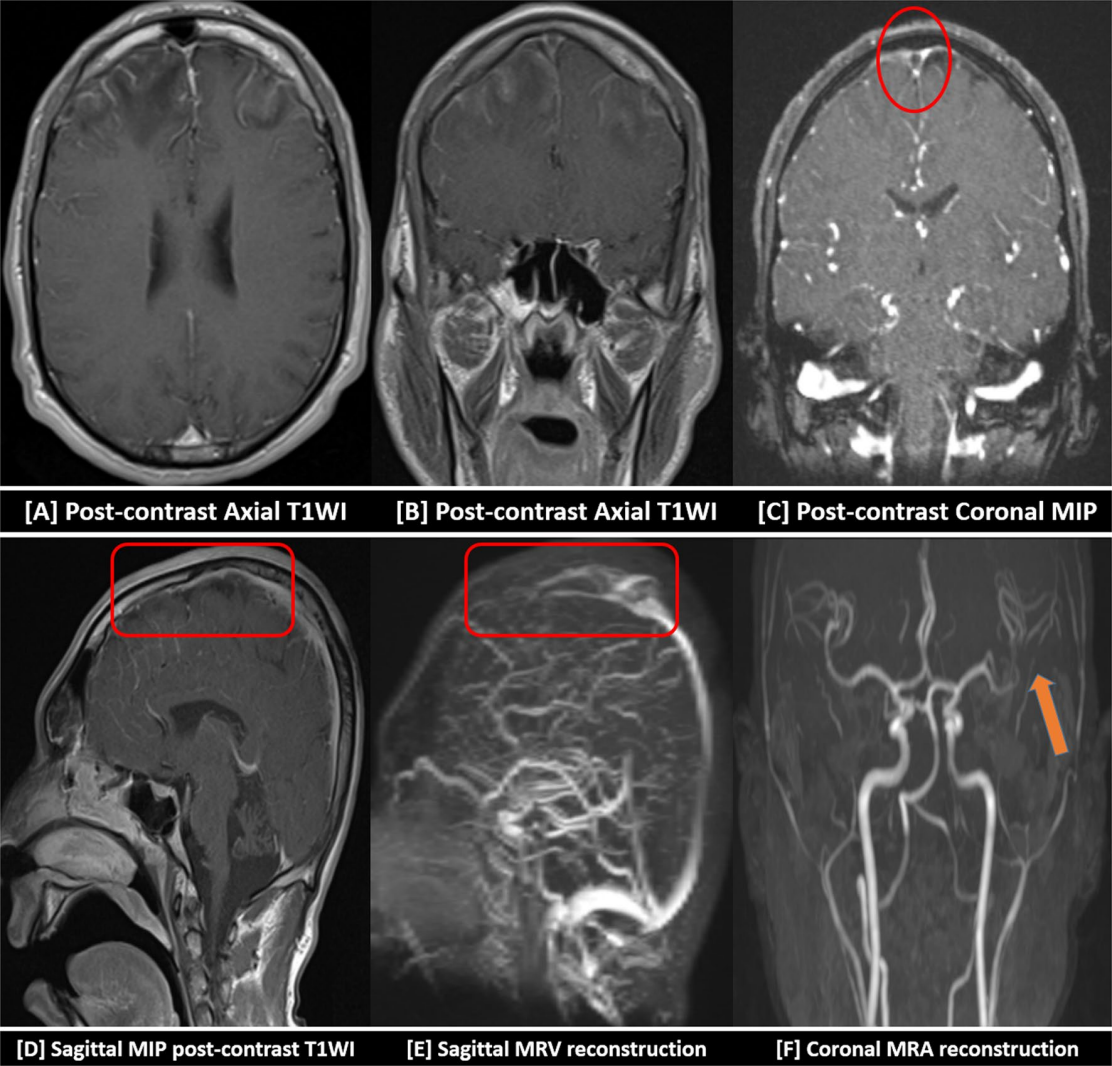
Contrast-enhanced MRI with MRV and MRA were performed for the above-mentioned patient (Fig. 6). [**A**, **B**] Patchy ischemic gyral enhancement was noted after IV contrast administration in T1-WI. [**C**] Central filling defect (delta sign) was noted in the superior sagittal sinus at the post-contrast coronal T1-WI with MIP reconstruction (red circle). [**D**] Long segment filling defect was seen at the post-contrast sagittal T1-WI with MIP reconstruction (red square). [**E**] This was also demonstrated in sagittal MRV reconstruction (red square). [**F**] MRA revealed attenuation of the lumen of the left M3 at the left Sylvian fissure as well as distal M4 cortical branches (orange arrow) … Final diagnosis was bilateral frontoparietal infarctions sequel to superior sagittal sinus thrombosis with hyper-acute hemorrhagic transformation. Additionally, left M3 and M4 attenuation was reported

- Non-enhanced brain CT examination was performed on all patients.
- Non-enhanced brain CT examination was performed alone on 192/262 (73%) patients. 188/262 (72%) patients of them showed hypo-dense areas of non-hemorrhagic infarctions (Fig. 2). Meanwhile, three patients showed hemorrhagic infarctions with mixed hypo and hyper-densities.
- Brain CTA examination was additionally performed on 6/262 (2%) patients. 5/6 patients of them showed attenuated MCA cortical branches. Meanwhile single patient of them showed complete left ICA and MCA occlusion. He had additional CTP and DWI-MRI examinations which showed diffusion-perfusion mismatch and a significant area of penumbra (Figs. 3, 4).
- Combined non-enhanced brain CT and closed MRI examinations were performed on 41/262 (16%). 34/41 patients of them showed single or multiple recent non-hemorrhagic infarctions expressing low T1 and high T2/FLAIR signal intensity with bright DWI signal and low ADC values denoting diffusion restriction. 2/41 patients of them showed hemorrhagic infarctions with SWI signal blooming. 5/41 patients of them showed MRI signs of vasculitis including cortical areas of high T2/FLAIR signal intensity in addition to patchy deep white matter similar lesions without diffusion restriction (Fig. 5). Regression up to a resolution of these vasculitic patches was encountered in the next follow-up examinations.
- Combined non-enhanced brain CT and closed MRI examinations with additional MRV examinations were performed for 15/262 patients (5%); six of them showed venous sinus thrombosis and hemorrhagic infarction with SWI signal blooming.
- Combined non-enhanced brain CT and closed MRI examinations with additional MRA and MRV examinations were performed for 8/262 patients (3%); two of them showed venous sinus thrombosis and hemorrhagic infarction with SWI signal blooming (Figs. 6, 7).

### [II] Pulmonary vasculopathy results

Pulmonary vasculopathy was encountered in 10/262 (3.5%) patients.

#### Clinically (Table 1)

5/10 (50%) patients clinically presented during the second week of infection, while 3/10 (30%) patients clinically presented during the third week of infection and 2/10 (20%) patients clinically presented during the fourth week of infection.

All patients were clinically presented with cough, dyspnea, and chest pain while 7/10 (70%) patients were feverish.

#### Radiologically

**Fig. 8:**
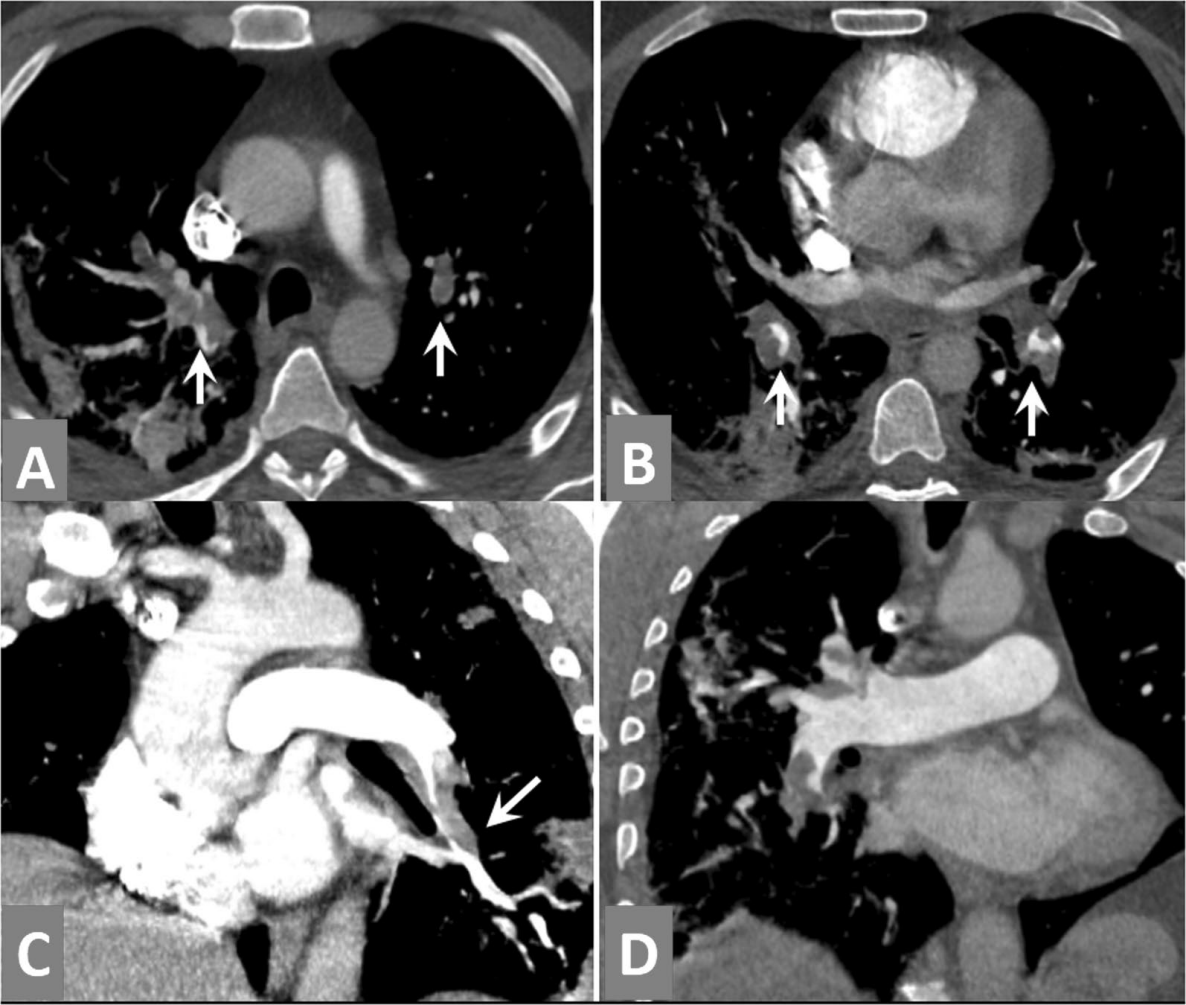
CT-pulmonary angiography (CTPA) examination for a 74-years-old COVID-19 male patient complained of severe chest pain and sudden deterioration of oxygen saturation at the third week of infection. [**A**, **B**] Axial CTPA images showing bilateral upper and lower lobar central contrast filling defects together with sub-pleural consolidative atelectatic patches. [**C**] Coronal CTPA image with MIP reconstruction showed left lower lobar large contrast filling defect extending to the peripheral arterioles with nearby sub-pleural wedge-shaped patch. [**D**] Coronal CTPA image showing similar right upper lobar changes … Massive pulmonary embolism and pulmonary infarctions

**Fig. 9:**
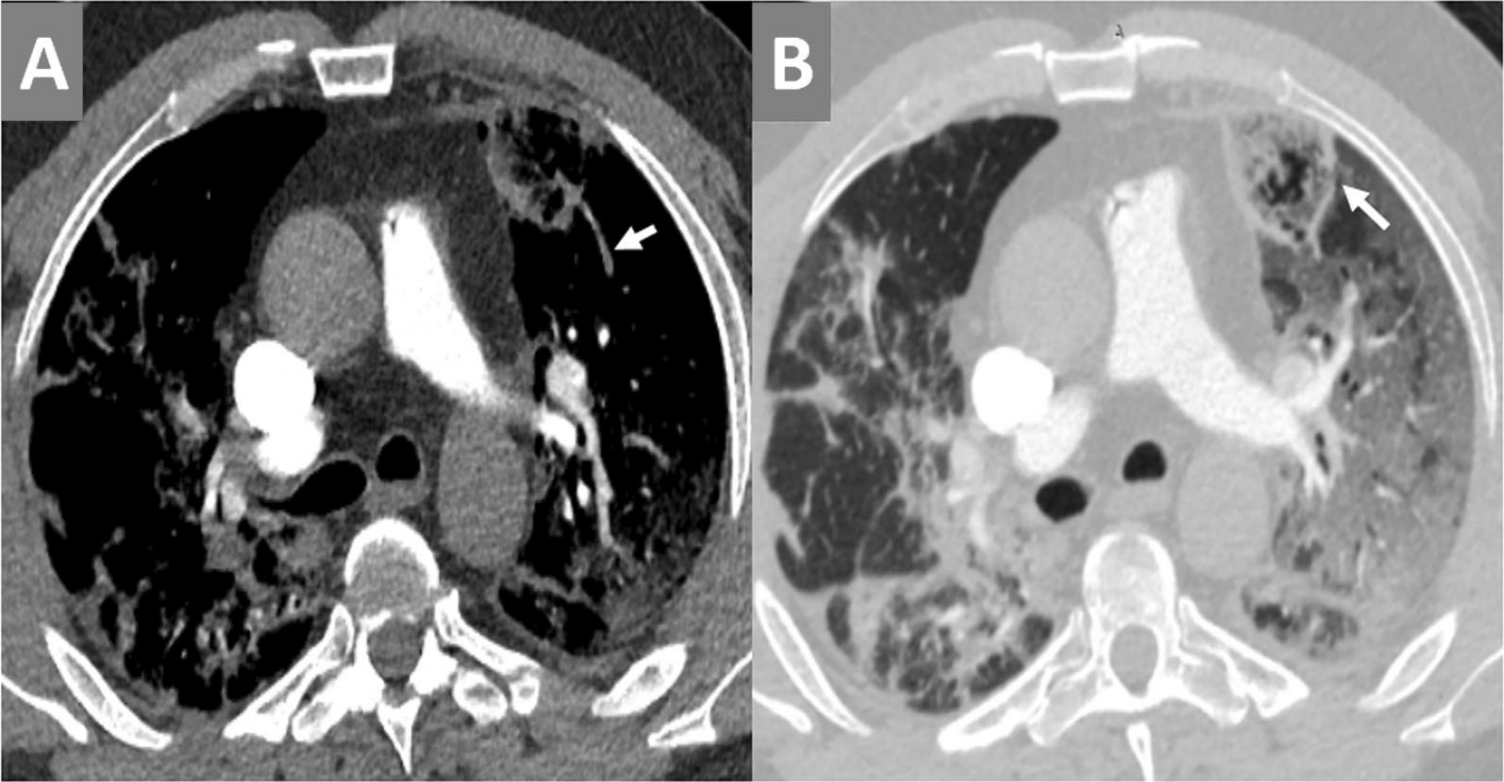
CT-pulmonary angiography (CTPA) examination for a 69-years-old COVID-19 male patient complained of severe chest pain and sudden deterioration of oxygen saturation in the second week of infection. [**A**] Axial CT image with MIP reconstruction showed left upper lobar peripheral acute pulmonary embolism (white arrow) and nearby sub-pleural wedge-shaped pulmonary infarction. [**B**] Axial CT image (lung window) showed the class (atoll-sign) of the pulmonary infarction (white arrow) and typical sub-pleural ground-glass bilateral patches of COVID-19 infection

**Fig. 10:**
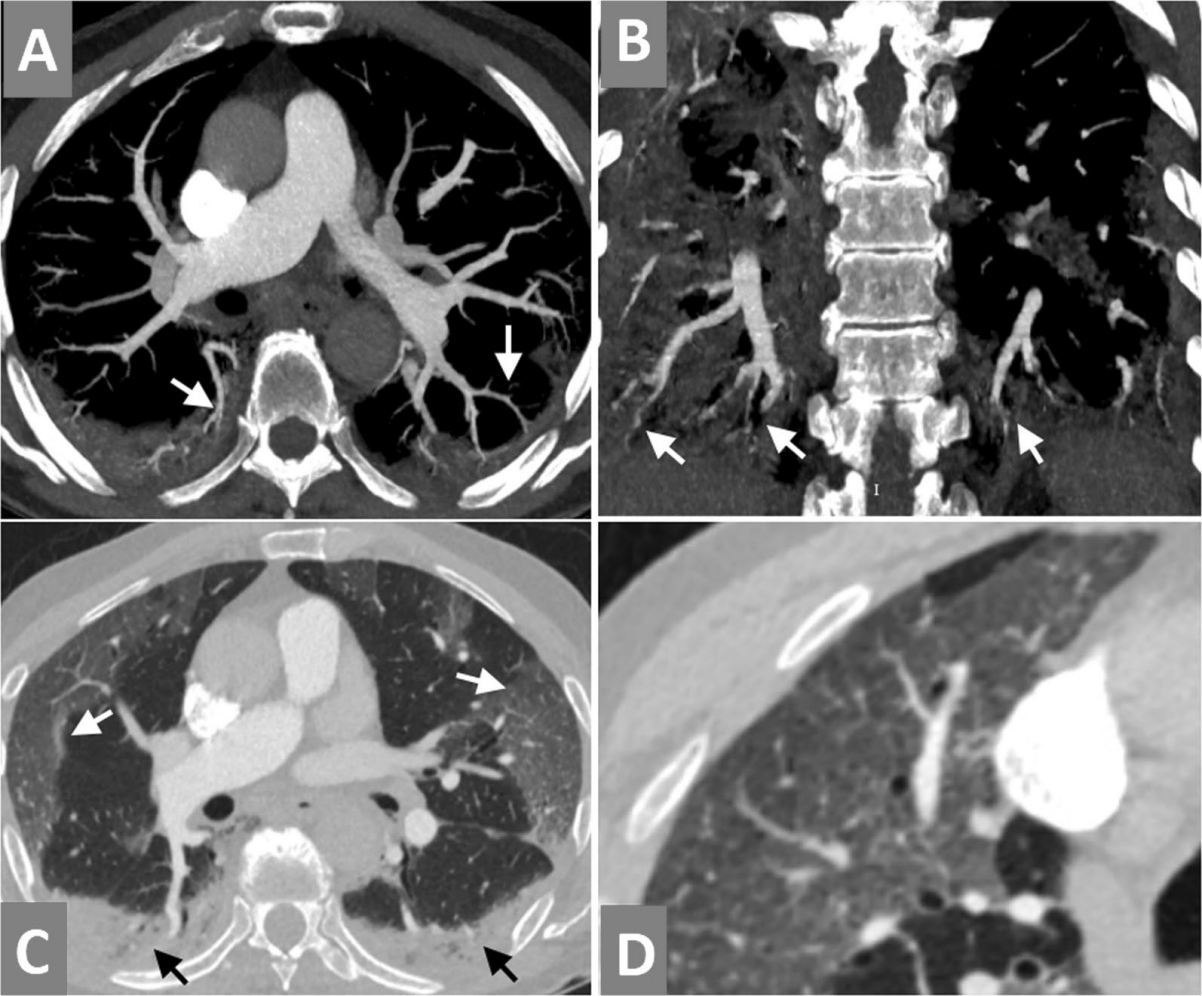
CT-pulmonary angiography (CTPA) examination for a 56-years-old COVID-19 male patient in the second week of infection. [**A**] Axial CTPA and [**B**] Coronal CTPA images showed bilateral basal dilatation of the peripheral pulmonary arterial tree reaching the pleural surface with a "loss of normal tapering" sign. [**C**] Corresponding axial CT image (lung window) showed bilateral sub-pleural ground-glass patches with exaggerated density at the basal gravitational lung zones. [**D**] Zoomed axial CT image (lung window) showed asymmetrical abnormal dilatation of pulmonary arterial branch inside the right upper lobar ground-glass patch … Pulmonary vascular enlargement (an indirect sign of pulmonary vasculopathy)

**Fig. 11:**
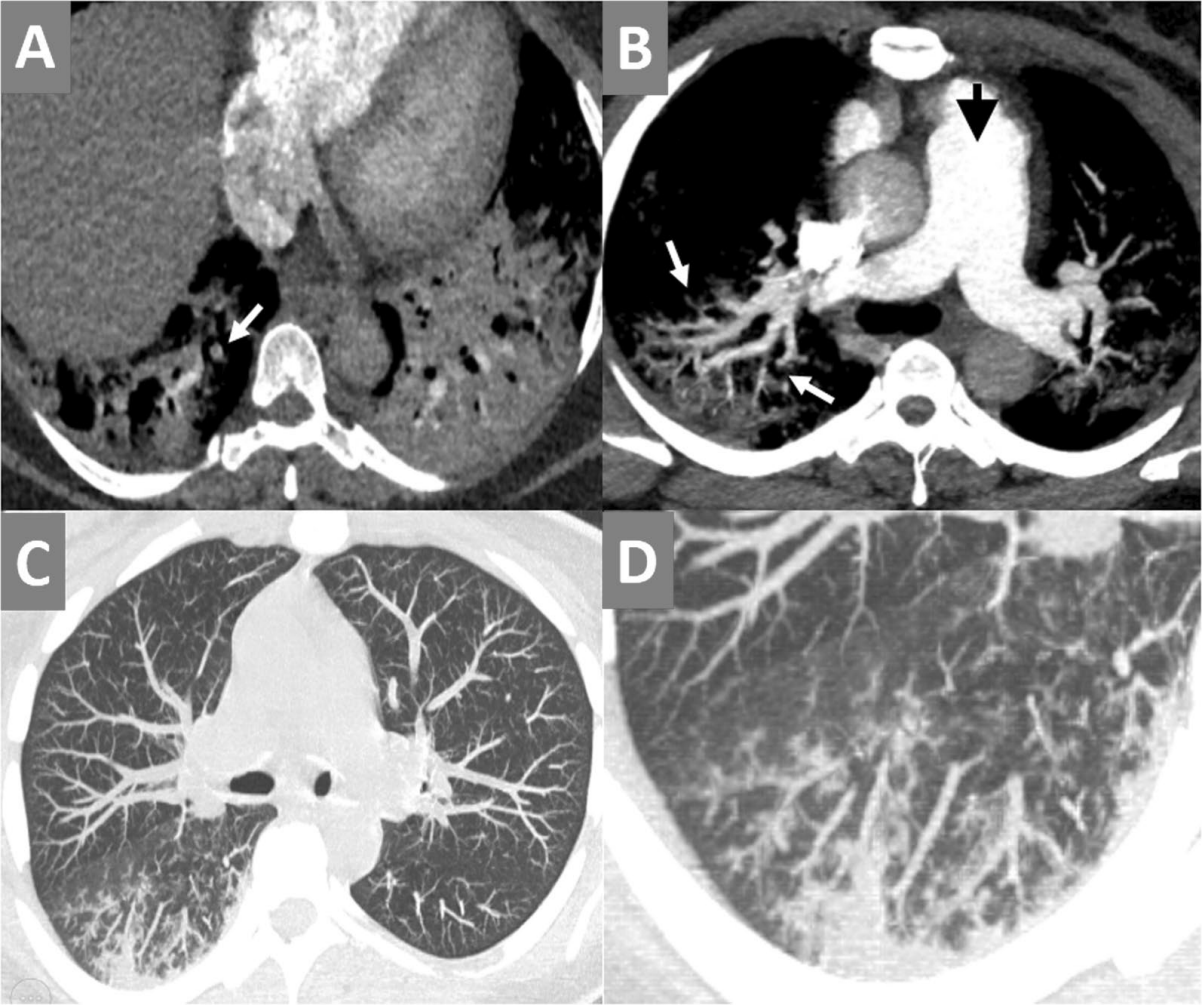
CT-pulmonary angiography (CTPA) examination for a 57-years-old COVID-19 male patient complained of severe chest pain and sudden deterioration of oxygen saturation in the second week of infection. [**A**] Axial CTPA image showed small contrast filling defect inside one of the right basal pulmonary arterial branches … Acute pulmonary embolism. This is together with bilateral basal consolidative and atelectatic patches. [**B**] Axial CTPA image showed dilatation of the pulmonary trunk exceeding the caliber of the nearby aorta (black arrow) referring to pulmonary hypertension. This is together with a right basal pulmonary vascular enlargement (white arrows). [**C**] Corresponding axial CT image with MIP reconstruction (lung window) and [**D**] zoomed image showing branching beaded nodular pattern continuous with the course of the pulmonary arterial tree … Vascular tree-in-bud sign (an indirect sign of pulmonary vasculopathy)

- In CTPA examinations, acute pulmonary embolism was found in 10/10 patients with central filling defects in either main or peripheral pulmonary arterial tree. 8/10 patients had additional pulmonary wedge-shaped infarcts (Figs. 8, 9).
- Two indirect CT signs of pulmonary vasculopathy were encountered; the first was the "pulmonary vascular enlargement" seen in 5/10 patients (Figs. 10, 11) and the other was the "vascular tree-in-bud sign" seen in 2/10 patients (Fig. 11).
- Lung CT-severity score 2 which corresponds to 26–50% lung involvement was dominant in 60% of patients.

### [III] gastro-intestinal vasculopathy results

GIT vasculopathy in CTA examinations of the abdominal aorta and mesenteric vessels was encountered in 7/262 (2.5%) patients.

**Fig. 12:**
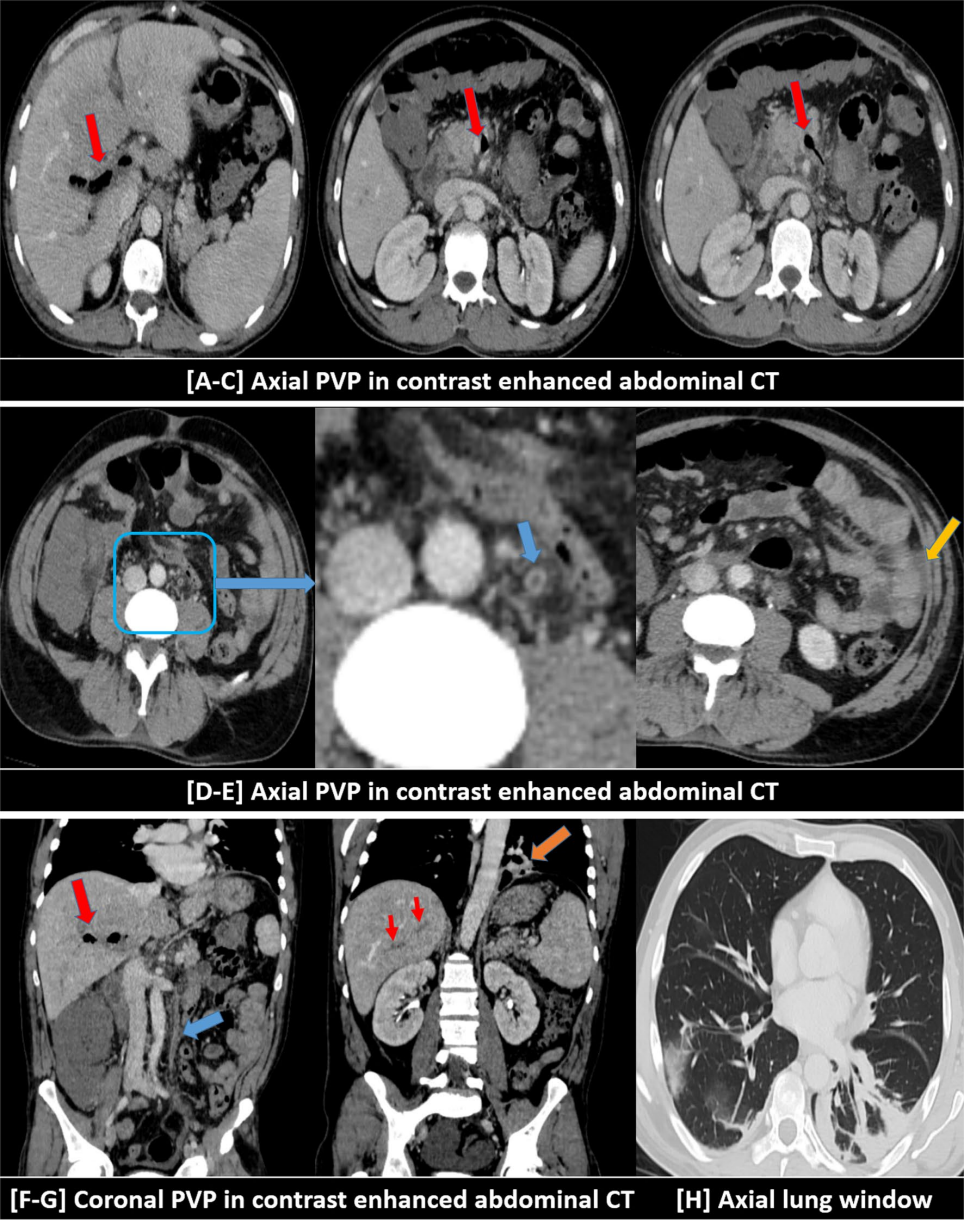
A 63-years-old male patient was proved for COVID-19 infection. He presented to the emergency unit during the second week of infection with severe abdominal pain. Mesenteric CT arteriography and venography were performed without oral preparation. [**A**–**C**] Axial images of abdominal CT at the venous phase revealed thrombosis of the portal venous system till the porto-mesenteric confluence and proximal part of the superior mesenteric vein (SMV) with intra-luminal air (red arrows). [**D**] Axial image of abdominal CT at the venous phase with zoomed images revealed thrombosis of the inferior mesenteric vein (IMV) (blue arrow). [**E**] Axial CT image showed mild mural edematous changes of the jujenal bowel loops with mild fluid collection in between denoting ischemic changes (yellow arrow). [**F**, **G**] Coronal images of abdominal CT at the venous phase revealed thrombosis of the portal vein with internal air (red arrows), thrombosis of the inferior mesenteric vein (blue arrow), and left basal pulmonary consolidative patch (orange arrow). [**H**] Axial image of the basal lung zones (lung window) revealed a right basal sub-pleural ground-glass patch and left basal consolidative atelectatic patch (typical for COVID19 infection)

**Fig. 13:**
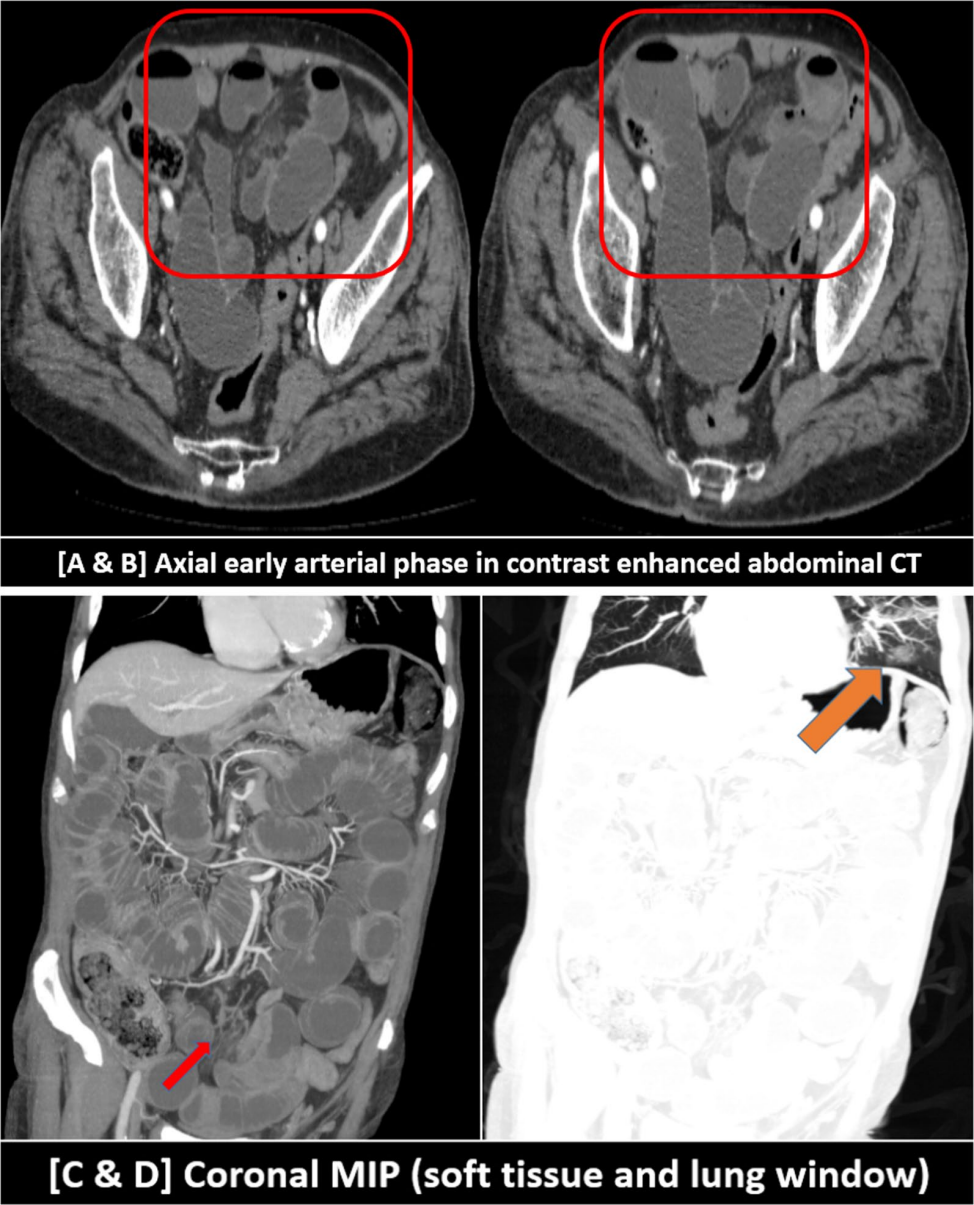
A 55-years-old male patient was proved for COVID-19 infection. He presented to the emergency unit during the second week of infection with severe abdominal pain and vomiting. Mesenteric CT arteriography and venography were performed without oral preparation. [**A**, **B**] Axial images of abdominal CT at the arterial phase revealed dilatation of the small bowel loops (ileus), meanwhile, some of the distal peripheral arcades of the superior mesenteric artery (SMA) are non opacified (red square) with stranding of the surrounding fat planes and minimal mural thickening of the supplied ileal loops. [**C**] Coronal image of abdominal CT at the arterial phase with MIP reconstruction demonstrated the severely attenuated distal SMA arcades inferiorly. [**D**] Coronal image (lung window) at the same left showed left basal ground-glass COVID-19 pneumonic patch

Intestinal ischemia and small bowel obstruction or ileus were found in 2/7 patients during the second week of infection with persistently positive PCR results, shooting D-dimer levels and initiated cytokine storm with elevated interleukin-6 (IL-6) level, as follows:

- 1/3 (33%) patients showed thrombosis of the portal venous system till the porto-mesenteric confluence and proximal part of the superior mesenteric vein (SMV) with intra-luminal air. He also showed thrombosis of the inferior mesenteric vein (IMV). There were mild mural edematous changes of the jujenal bowel loops with mild fluid collection in between denoting ischemic changes in addition to small bowel dilatation and ileus (Fig. 12). The viral lab profile of this patient was negative and CT did not show signs of liver cirrhosis. The chest CT images showed positive lung involvement.
- 1/3 (33%) patients showed non-opacification of the distal peripheral arcades of the superior mesenteric artery (SMA) at the pelvic region with minimal mural thickening of the related ileal bowel loops and stranding of the surrounding fat planes. Small bowel dilatation and ileus were also noted (Fig. 13). No significant arterial atherosclerotic changes were encountered.

On the other hand and without respiratory complaint, the examination of 1/7 patients showed non-opacification of the peripheral superior mesenteric arterial arcades. Long segments of small bowel loops were dilated and gas-filled with minimal mesenteric fat stranding denoted early ischemic changes. The chest CT scans were free. This patient was admitted because of severe abdominal pain. The routine pre-admission PCR testing for COVID-19 was strikingly positive. The D-dimer level was shooting. Only mild atherosclerotic changes were noticed in CT images which could not explain this condition. COVID-19 mural endothelial injury with ischemic necrosis was given as a reasonable clinical alternative diagnosis.

Mixed upper and lower GIT bleeding was encountered in 4/7 patients during the third week of infection after relapse of fever and deterioration of respiratory symptoms. All are presented by hematemesis and melena. CT examinations in collaboration with urgent upper GI-endoscopy showed signs of severe gastritis with retained intra-luminal blood. The four patients died shortly from severe anemia and multi-system organ failure.

### [IV] Peripheral limb vasculopathy results

Lower limb ischemic and arterial occlusions were found in Duplex ultrasound and CTA examinations in 3/262 (1%) patients.

**Fig. 14:**
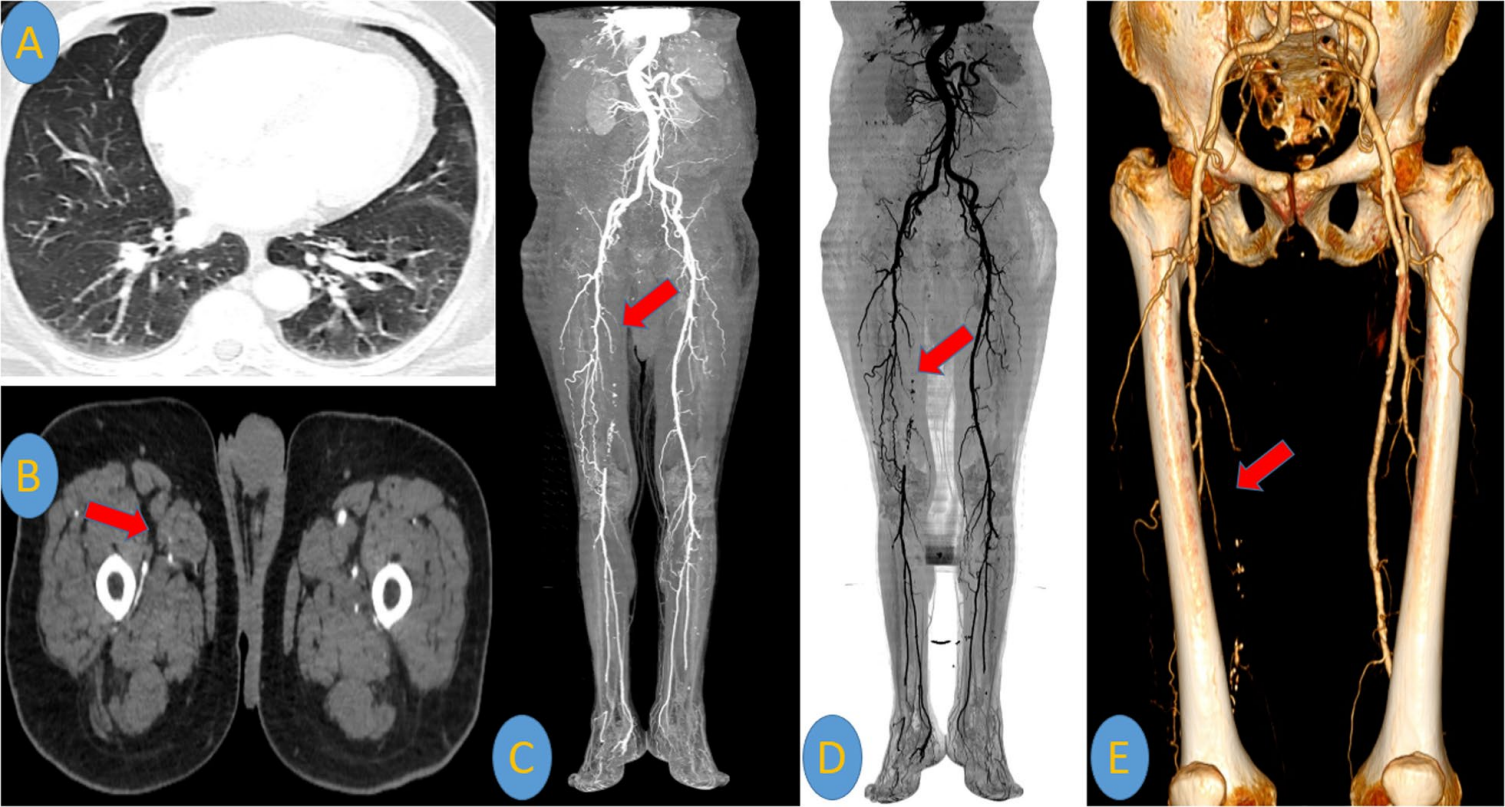
A 67-years-old male patient was proved for COVID-19 infection. He presented to the emergency unit three weeks after infection with severe right leg pain and coldness. [**A**] Axial image of chest CT revealed faint bilateral sub-pleural COVID-19 pneumonic ground-glass patches. CTA of the lower abdominal aorta and peripheral lower limbs was performed. [**B**] Axial image of CTA revealed occlusion of the right superficial femoral artery (SFA). [**C**–**E**] Volume rendering (VR) reconstruction demonstrated this SFA occlusion. [**C**] Surface shaded VR image, [**D**] Negative surface shaded VR image, [**E**] Colored VR image (red arrows)

**Fig. 15:**
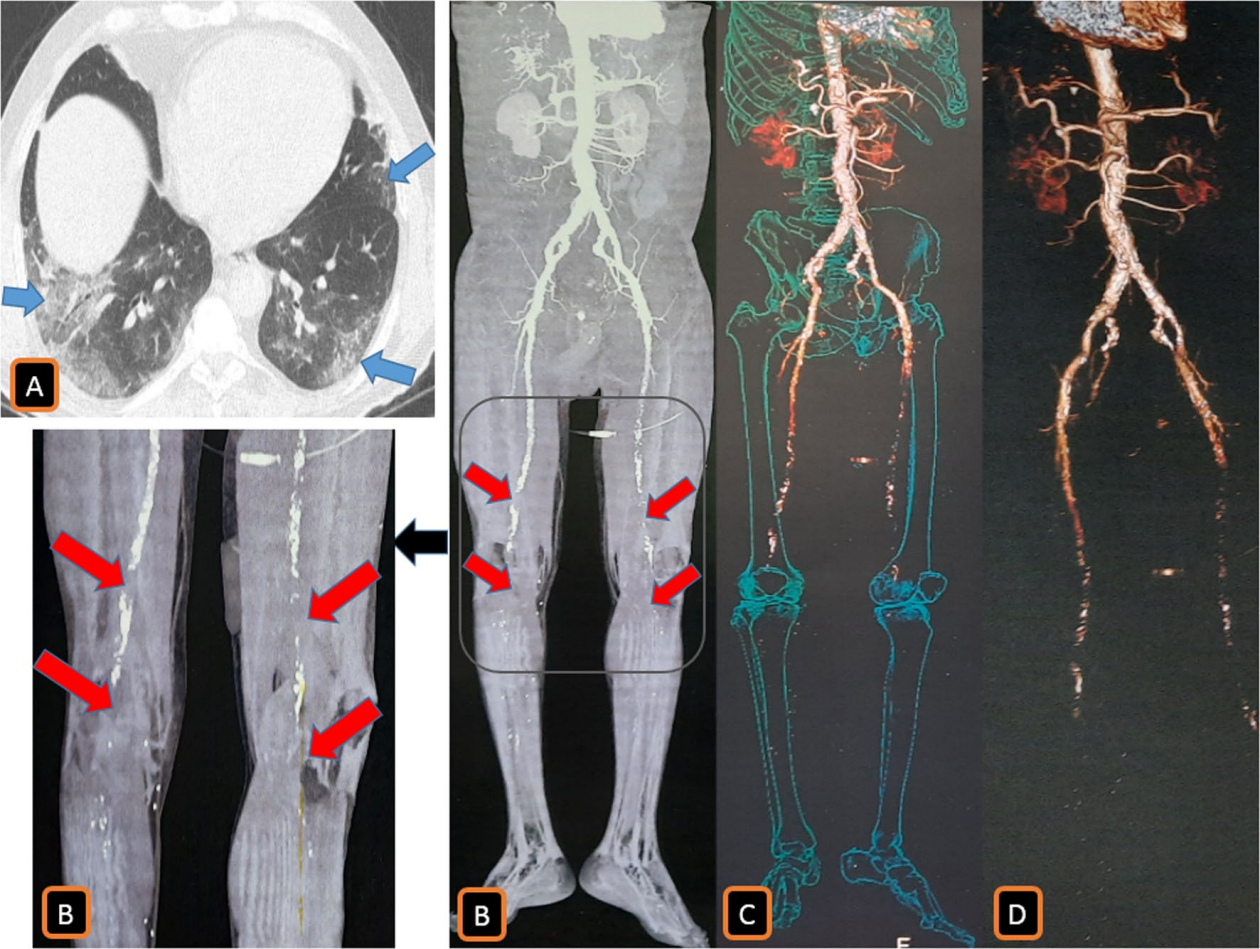
A 53-years-old male patient with a history of peripheral atherosclerosis. He presented to the emergency unit with acute severe bilateral leg pain and coldness. No chest symptoms were encountered. [**A**] Chest CT was urgently performed before admission, it revealed bilateral sub-pleural typical COVID-19 pneumonic ground-glass patches (blue arrows). CTA of the lower abdominal aorta and peripheral lower limbs was performed. [**B**–**D**] Surface shaded and colored VR images showed bilateral acute SFA and popliteal artery occlusion on top of heavy atherosclerotic changes with non-opacification of the popliteal trifurcation of infra-genicular arteries bilaterally (red arrows). PCR test result for COVID-19 infection was revealed to be positive and the D-dimer level was shooting (5125 ng/ml)

- 2/3 (67%) patients of them presented to the emergency unit during the second and third week of infection with severe leg pain and cyanosis. CTA examinations showed unilateral superficial femoral arterial (SFA) occlusion (Fig. 14).
- Meanwhile, 1/3 (33%) patient of them with a history of atherosclerosis presented to the emergency unit with bilateral severe leg pain and cyanosis. No chest symptoms were encountered. CTA showed bilateral acute SFA and popliteal artery occlusion on top of heavy atherosclerotic changes with non-opacification of the popliteal trifurcation of infra-genicular arteries bilaterally. Chest CT was routinely performed before admission and showed typical COVID-19 pneumonic patches. PCR test result for COVID-19 infection was positive and D-dimer level was shooting (5125 ng/ml) (Fig. 15).

### [V] Multi-system vasculopathy

Mixed involvement between the above-mentioned different systems of vasculopathies was encountered in 30/282 patients (11%).

### [V] Post-management coagulopathy

Duplex ultrasound examinations were performed on 57/282 (20%) patients during their hospital and ICU stay. 39/282 (13.8%) patients developed peripheral deep venous thrombosis (DVT) with prolonged recumbence while 28/282 (10%) patients developed jugular vein thrombosis sequel to prolonged catheterization.

## Discussion

The pulmonary parenchymal complications and respiratory failure are initially considered the major cause of mortality after COVID-19 infection. But when autopsies evidenced widespread vasculopathy, this directed the clinicians towards different pathophysiology for the development of respiratory failure in COVID-19 patients from the 'conventional' post-infectious adult respiratory distress syndrome, which is COVID-19 related vasculopathy [14].

### Assortment of the previous relevant research in Egypt

After two years of the pandemic, a long-term collective Egyptian experience is provided in this study on 282 patients with different multi-systemic imaging findings of COVID-19 vasculopathy. Additional to the frequent thrombo-embolic disorders, it included the indirect CT signs of pulmonary vasculopathy and collected other rare complications such as cerebral vasculitis and GIT bleeding.

The review of the previous literature regarding the COVID-19 vascular complications in Egypt revealed few types of research. Conversely to this study, almost all of them were short-term studies that were conducted on a fewer number of patients and targeted only the thrombo-embolic category of vascular complications. They were as follows:

Omar et al. [15] targeted the multi-systemic thrombo-embolic complications of COVID-19 infection but without other vasculopathy disorders. It was a four-month study that ended earlier in July 2020. The incidence of thromboembolic complications was 124/1245 (10%) which was near to this study (8%). Saleh et al. [16], and Abdelzaher et al. [17] studied only the vascular and non-vascular neurological complications of COVID-19 infection. The former included 70 patients over 14 months till July 2021 and the latter included 135 patients over six months till December 2020. Abdelmohsen et al. [18] studied the vascular and non-vascular gastrointestinal complications of COVID-19. It included 30 patients over three months in 2020. Yassin et al. [19] studied the pulmonary vascular complications of COVID-19. It included 40 patients over four months till July 2020.

In this study, the mean age of the included patients was 68 years. This could be explained by the higher incidence of vascular comorbidities among the older age groups and the loss of the protective role of estrogen in post-menopausal females. It was near that estimated by Abdelzaher et al. [18] which was 63 years. On the other hand, both exceeded that estimated by Saleh et al. [16] which was 43 years.

In this study, the incidence of COVID-19 vascular complications in this study was higher in men. This could be explained by the higher incidence of smoking in men and also the presence of the protective role of estrogen in females. This was in keeping with the previous research [16–19].

In this study, hypertension was the most common comorbidity in this study. This was matching with Saleh et al. [16]. On the other hand, diabetes mellitus was the most common comorbidity in Omar et al. [15].

Agreeing with Yassin et al. [19], COVID-19 vasculopathy was most commonly seen in the second week after infection with a strong to relation elevated D-dimer levels. On the other hand, Omar et al. [15] reported them earlier during the first week of infection.

In this study, the neuro-vascular complications were the most common vasculopathy (93%). Conversely, Omar et al. [15] found them only in 25.8% of their patients while the pulmonary vascular complications were most commonly seen in 45.2% of patients.

Agreeing with Saleh et al. [16], and Abdelzaher et al. [17], the ischemic infarctions were the most common neurovascular complications and a disturbed level of consciousness was the most common clinical presentation. Meanwhile, the presence of hemorrhagic insults and vasculitis was infrequent.

In this study, ischemic small bowel changes were the most common gastrointestinal vascular complication. This matches Abdelmohsen et al. [18].

### Assortment of the previous relevant studies outside Egypt (worldwide)

This study disagreed with the previous research by Vadvala et al. [20] which reported that the bleeding complications with or without contrast extravasation were most commonly seen in 71% of included patients, while thromboembolic and ischemic complications were seen only in 17.8% of patients. Other worldwide researchers also disagreed with this, including Shah et al. [21], Lodigiani et al. [22], Lee et al. [23], O'Shea et al. [24], Abdelmohsen et al. [25].

### Limitations and strengths of the study

The study may be limited by the absence of statistical correlation analysis between lung volumetry and vasculopathy. The authors recommend further future studies for this relation using a large control group or meta-analysis.

The large number of included patients over long duration interval (around 2 years) particularly in Egypt side-by-side with the clinical presentation are the main merits of this study. The authors believed that sharing experiences about the COVID-19 pandemic among different parts of the world after the past 2 years is extremely important to add to the early published literature and to better understand the disease and learn from this experience in the future.

## Conclusions

Multi-system vasculopathy was a serious complication of COVID-19 which impacted the patients' morbidity and mortality. An Egyptian experience about the COVID-19 vasculopathy during the past two years of the pandemic was provided. It encountered the different modalities and imaging techniques for the diagnosis of cerebrovascular, pulmonary, gastrointestinal, and peripheral arterial COVID-19 vascular complications.

## Data Availability

The datasets used and/or analyzed during the current study are available from the corresponding author on reasonable request.

## Abbreviations

Covid-19: Novel coronavirus disease (2019)
CT: Computed tomography
CTA: Computed tomography with angiography
MPR: Multi-planar reconstruction
MRI: Magnetic resonance imaging
MRA: Magnetic resonance arteriography
MRV: Magnetic resonance venography
MRP: Magnetic resonance perfusion
